# Age-related changes to motor synergies in multi-joint and multi-finger manipulative skills: a meta-analysis

**DOI:** 10.1007/s00421-019-04216-4

**Published:** 2019-08-31

**Authors:** Mohsen Shafizadeh, Ali Sharifnezhad, Jonathan Wheat

**Affiliations:** 1 Faculty of Health and Wellbeing, Sheffield Hallam University, Sheffield, S10 2BP UK; 2 Department of Sport Biomechanics, Sport Sciences Research Institute, Tehran, Iran

**Keywords:** Synergy, Ageing, Grasping, Reaching

## Abstract

**Purpose:**

The aim of the current meta-analysis was to examine the extent to which there are differences in upper extremity motor synergies across different age groups in manipulative tasks.

**Methods:**

The studies that used the uncontrolled manifold method to examine the effect of age on motor synergies in multi-joint and multi-finger tasks were selected. Sixteen relevant studies from 1154 articles were selected for the meta-analysis—4 and 12 studies considered multi-joint kinematics and multi-finger kinetic tasks respectively.

**Results:**

The results of the meta-analysis suggested reduced strength of synergies in multi-finger task in older adults, but this was not the case for synergies in multi-joint task. Part of this age-related difference in finger function is related to the increased variability in total force in grasping tasks. However, reductions in the strength of multi-finger synergies in hand functions following ageing appear to depend on the characteristics of the task.

**Conclusions:**

These findings indicate that the cooperation among fingers to stabilise the total required force to apply for grasping and other fine motor skills is less efficient in older adults that might affect the quality of manipulative tasks.

## Introduction

Participation in activities of daily living (ADL) has a significant impact on the mental health and physical fitness of older adults (Hasselkus 2002; Wilcock 1998). Broadly, ADLs can be personal—such as dressing, bathing and eating—and instrumental—such as house maintenance, community mobility and so on (Kempen and Suurmeijer 1990; Fisher 1997), but they can be further categorised into postural, locomotor and manipulative skills (Gallahue et al. 2012).

The upper limbs play an important role in manipulative tasks as they are usually involved in reaching, catching and grasping (Verrel et al. 2012). These movements require the coordination of multiple body segments, often with the goal of stabilising performance variables such as total force in grasping and wrist position for aiming (Latash and Anson 2006). Instead of eliminating the available degrees of freedom, it has been suggested that the central nervous system (CNS) organises them in functional units known as motor synergies to effectively control the limb movement to achieve the desired outcome (Gelfand and Latash 1998).

Generally, motor synergies have an important role to stabilise the performance variable against internal and external perturbations (Latash et al. 2007). For example, in catching an important performance variable is the accuracy of end-effector position. The main task of the CNS is to move the multi-segment unit—including shoulder, elbow and wrist—towards the target to complete the task with low end-effector position variability. If the target is moving unpredictably, the segments in the synergy are re-shaped accordingly to maximise end-effector accuracy. Motor abundance theory (Gelfand and Latash 1998) suggests that providing motor variability is an important role of the CNS to ensure that adjustments occur in response to changing environmental and task demands (Latash 2012).

One method to quantify the motor synergies is the uncontrolled manifold (UCM) model (Scholz and Schöner 1999). The model is based on the association between variability in a performance variable (e.g. end-effector position) and variability in elemental variables (e.g. joint angles). Two types of variability in the elemental variables are possible: goal-equivalent variance (GEV) and nongoal-equivalent variance (NGEV). GEV is variability in the elemental variables that have no effect on the performance variable. On the other hand, NGEV is variability in the elemental variables influencing the performance variable. The stability of the motor system against any perturbation is determined by the ratio of GEV–NGEV (Latash et al. 2007). Larger ratios represent stronger synergies. In other words, the accuracy of end-effector movements and stability of the performance variables are two important characteristics of motor synergies that have significant roles in manipulative skills. Accuracy is determined by the trial-to-trial variability in a target performance outcome (e.g. spatial errors), whereas the stability emphasises on coordination variability among the elemental variables that stabilises the performance variable in successive attempts (Gelfand and Latash 1998).

Motor synergies are required for effective upper-limb function for older adults. Some studies reported a shift from synergic to element-based control due to ageing. Synergic control implies that movements are controlled collectively through activation of cortical neurons that work as a unit. This harmonic neural control is lost with ageing, which results in a less synergic, or more element-based, control (Gorniak et al. 2011). Structural, physiological and sensory-motor changes have been implicated as potential mechanisms for reduced motor synergies in the upper-limb function of older people (Rodgers and Evans 1993; Francis and Spirduso 2000; Cole 1991; Hayase et al. 2004) and people with neurological conditions such as Multiple Sclerosis (Jo et al. 2017), Down Syndrome (Latash et al. 2002a, b) and Parkinson's disease (Jo et al. 2015). Older adults exhibit subclinical dysfunctions in the central and peripheral nervous systems such as increased muscle co-activation, smaller muscles, fewer muscle fibres, impaired intercortical inhibition (Beijersbergen et al. 2013; Faulkner et al. 2007; Thompson 2009), emergence of larger and slower motor units and a reduced ability to produce muscle force (Larsson and Ansved 1995; Cole et al. 1999). Subsequently, this could affect the quality of upper-limb movements in manipulative tasks that require the fingers to grip at the same time as keeping the arm steady—such as drinking, eating, writing, holding, and dressing (Grabiner and Enoka 1995). For example, older adults exhibit excessive grip forces and a reduced ability to maintain low grip force (Cole et al. 1999; Lindberg et al. 2009). In addition, older adults show more variability in hand path than young adults in multi-joint reaching tasks (Dutta et al. 2013) and execute the movements slower, less accurately and less steady than young adults (Bock 2005; Heuer and Hegele 2008; Buch et al. 2003).

However, contradictory studies have reported that motor synergy is preserved in older adults during reaching (Greve et al. 2017; Lee et al. 2007; Krüger et al. 2013; Xu et al. 2013) and grasping (Singh et al. 2013; Skm et al. 2012). The contradictory findings were explained by those control mechanisms that are independent of motor flexibility (Greve et al. 2017) and depend on the nature of task constraints (Krüger et al. 2013; Xu et al. 2013). For example, motor synergies are preserved for longer in tasks that are similar to ADLs (Skm et al. 2012; Xu et al. 2013), in rapid reaching task (Greve et al. 2017) and in the multiple-task conditions (Krüger et al. 2013) than laboratory and artificial tasks.

To elucidate the age-related changes in upper-limb motor synergies, the aim of this meta-analysis was to review the studies that have compared the motor synergy index and its variance components (GEV and NGEV) between young and older adults, with consideration of the influence of task constraints. More specifically, we separated our data synthesis into studies that examined synergies in multi-joint and multi-finger tasks that are required for either reaching or grasping. Reaching and grasping require different control mechanisms. Reaching movements involve proximal segments for arm transportation and distal segments for positioning and orientation of the end-effector (Jeannerod 1999). The main challenge in grasping task is to covary finger forces to stabilise total force production (Latash et al. 2007). Therefore, the current meta-analysis study addressed the two main questions: (1) do motor synergies and the associated variance components differ between young and older adults? (2) do age-related changes in motor synergies depend on the nature of the task?

## Methods

### Eligibility criteria

Studies that met the following criteria were included in this meta-analysis: (1) cross-sectional or pre-post (independent groups) research designs. (2) The sample included both adults (20–40 years) and older adults (> 65 years). (3) Manual task experiments included multi-joint task or multi-finger task. (4) The UCM method was used for the analysis. (5) Articles were peer reviewed and published in English between 2000 and 2018. Studies were excluded if they were case-study and non-peer reviewed articles and did not report any index for kinematic synergies and kinetic synergies.

### Search strategy and study selection

The following databases were searched: Cumulative Index to Nursing and Allied Health Literature (CINHAL), MEDLINE, Health Source: Nursing/ Academic Edition (HSNAE), SPORTDiscus, Scopus, Pubmed, Cochran Library and Allied and Complementary Medicine Database (AMED). The search strategy involved four steps, with a combination of two search terms used at each step. Step 1: "uncontrolled manifold" AND "ageing", step 2: "uncontrolled manifold "AND "older adults", step 3: "multi-joint coordination" AND "ageing", and step 4: "multi-joint coordination" AND "motor synergy" AND "older adults". Each time the combined terms search brought new studies; some were already included in our study and some were excluded from the final list of studies. Abstracts and full texts were screened by MS and AS to ensure that they met the inclusion criteria.

### Data extraction process

A spreadsheet was created to sort the studies according to the main inclusion criteria. Studies were organised in a Microsoft Excel worksheet according to methodological, task and research outcome information. The information on methods was sample size, age groups, task setting and synergy assessment methods.

### Synthesis of results

A meta-analysis was performed to calculate the pooled effect size (ES) for the synergy index and variance components (GEV and NGEV) for the differences between groups of young and older adults. A random-effect model was used at a 95% confidence interval using Cochran's *Q* test, with *I*^2^ statistics as indices of heterogeneity. A random effects model also accounts for differences in variability across studies by weighting each standardized effect on the basis of its standard error. The *Q* statistic is the sum of squares of the weighted mean standardized effect of each study within each variable (synergy index) divided by the overall weighted mean standardized effect for that variable.

Standardized effects indicate the magnitude of the effect of an independent variable, regardless of sample size. Standardized effects were calculated for each variable as the difference between group means (e.g. young and older adults) divided by the group pooled standard deviation. Meaningfulness was determined by Cohen’s classification (Cohen 1988): a standardized effect size of less than 0.2 was considered trivial, 0.2–0.5 was considered small, of 0.5–0.8 was considered moderate and above 0.8 was considered large. There were three dependent variables in this meta-analysis: motor synergy index, GEV and NGEV. Multiple meta-analyses were carried out including multi-joint (kinematic) tasks, multi-finger (kinetic) tasks, overall hand synergies (combination of both kinematic and kinetic studies), and groups of kinematic and kinetic synergies based on the unit of measurement.

All statistical analyses were conducted in Review Manager version 5.3.3 (Nordic Cochrane Centre). The two-tailed statistical significance level was set at *p* < 0.05.

### Study quality assessment

The Newcastle–Ottawa Quality Assessment Scale for cohort studies (Wells et al. 2005) was used to assess the study quality. The scale has eight items and three subscales including selection (four items), comparability (one item) and outcome (three items). The "selection subscale" assesses the quality of a study in terms of the representativeness of the selected participants, whether the group was non-exposed, the source of access to the sample and blindness. The "comparability subscale" mainly assesses the control of confounding factors. The "outcome subscale" assesses the method of data collection such as design, number of data collection sessions, and the survival rate in follow-up tests. The possible total score in each study ranges between 0 and 9. MS and AS screened the full texts and assessed their quality independently using all the above-mentioned items and an average score was reported. Discrepancies in quality rating were resolved by discussion. If consensus was not reached, a third reviewer (JW) was consulted.

## Results

### Search results

**Fig. 1:**
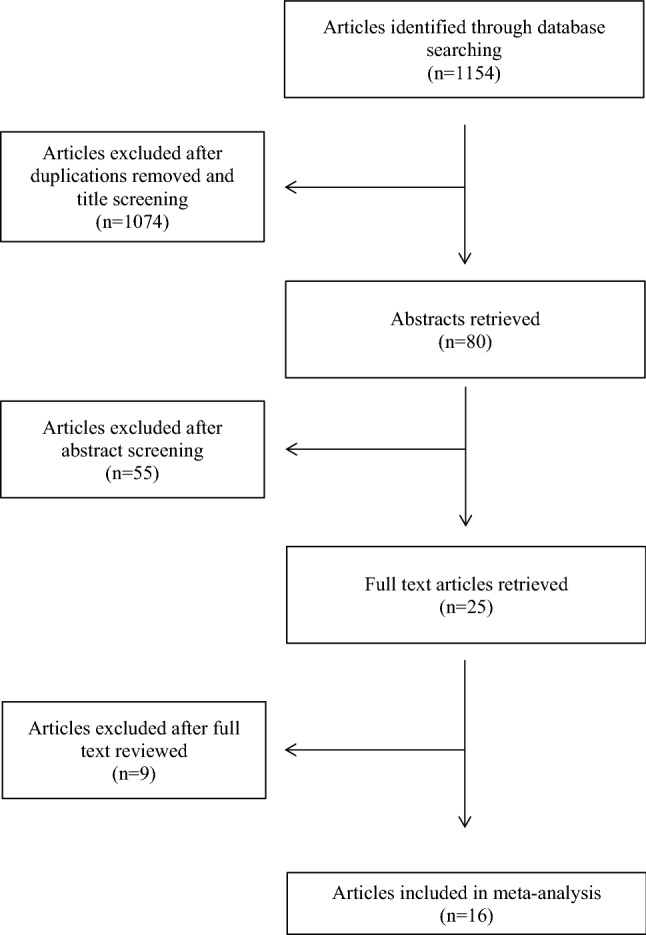
Flow diagram of selection of studies focusing on motor synergies in upper limbs

The search results yielded 1154 articles that reported synergies metrics. More specifically, the searches with a combined terms "uncontrolled manifold" AND "ageing" resulted in 687 articles. The combination of "uncontrolled manifold "AND "older adults" resulted in additional 392 articles. The combination of "multi-joint coordination" AND "ageing" and "multi-joint coordination" AND "motor synergy" AND "older adults resulted in 57 and 18 articles, respectively (see Fig. 1). After reading the titles, 1074 articles were excluded because they were case studies, were published in non-peer reviewed journals or did not report any index for kinematic synergies and kinetic synergies. Twenty five duplicate articles were removed. The abstracts of 80 articles were reviewed and only 25 articles were included. Studies that did not report any clear metrics in the text, or only had one participant group, were excluded after retrieving the full text (*n* = 9). Finally, 16 articles were selected for meta-analysis. There were 4 articles on multi-joint task (Dutta et al. 2013; Krüger et al. 2013; Verrel et al. 2012; Xu et al. 2013) and 12 articles on multi-finger task (Gorniak et al. 2011; Kapur et al. 2010b; Olafsdottir et al. 2007a,2007b; Park et al. 2011,2016; Shim et al. 2004; Shinohara et al. 2004; Singh et al. 2013; Skm et al. 2012; Solnik et al. 2012; Wu et al. 2013). Important information regarding the selected studies such as samples, models of synergies (kinematic/kinetic) and experimental tasks is presented in Table 1.

**Table 1:** The main characteristics of participants, synergy models and task

Studies	Quality score	Adults	Older adults	Types of synergy	Synergy model	Task
Dutta et al (2013)	6	Male and female (*n* = 11) healthy adults (27 ± 11 years); right-handed	Male and female (*n* = 10) healthy adults (67 ± 5 years); right-handed	Multi-joint kinematic	UCM model included a 10 Dofs elemental variable (clavicle, shoulder, elbow and wrist) and one performance variable (hand position); *V*_UCM_, *V*_ORT_ and R_*V*ucm/*V*ort_ were reported	Reaching and aiming to a target on a touch screen; fast and accurate; no reaction time requirement; right/left hands under certain and uncertain target conditions; 120 trials per condition; *only data from "dominant hand" and "certain the study*
Gorniak et al (2011)	9	Male and female (*n* = 13) healthy adults (23 ± 3 years); right-handed	Male and female (*n* = 9) healthy adults (78 ± 3 years); right-handed	Multi-finger force kinetic	UCM model included force variability among four fingers (elemental variable) to stablise a performance variable (grip force); Δ*V* was reported	Moving a handle to a visual target by applying fingers grip forces; subjects were instructed to move the handle quickly and accurately 0.30 m vertically to the visual target; 15 trials were completed
Kapur et al (2010a, b)	9	Male and female (*n* = 8) healthy adults (27.5 ± 3.7 years); right-handed	Male and female (*n* = 8) healthy adults (76.4 ± 2.5 years); right-handed	Multi-finger force kinetic	UCM model included force variability among four fingers (elemental variable) to stablise a performance variable (total force); *V*_UCM_, *V*_ORT_ and Δ*V* were reported	Finger pressing task in 4-fingers and 1-finger conditions; pressing the fingers in different directions (downward, backward, downward-left); online feedback from the force magnitude (MVC%); 15 trials in Four-finger pressing and 3 trials in 1-finger pressing; *only data from "Four-finger pressing" and "force magnitude" were used in this study*
Kruger et al. (2013)	6	Male and female (*n* = 11) healthy adults (25.5 ± 3.4 years); right-handed	Male and female (*n* = 11) healthy adults (66.3 ± 3.1 years); right-handed	Multi-joint Kinematic	UCM model included a 7Dofs elemental variable (shoulder, elbow and wrist) and one performance variable (hand position); *V*_UCM_, *V*_ORT_ and *R*_*V*ucm/*V*ort_ were reported	Reaching and grasping an object; fast and accurate; no reaction time requirement; target location was changed (L/R/M); 120 trials
Olafsdottir et al. (2007a, b)	8	Male and female (*n* = 12) healthy adults (26 ± 3 years); right-handed	Male and female (*n* = 12) healthy adults (77 ± 4 years); right-handed	Multi-finger force kinetic	UCM model included force variability among four fingers (elemental variable) to stablise a performance variable (total force); Δ*V* was reported	The subjects were asked to produce a ramp pattern of force from 0–25% of MVC over 5 s by pressing down with four fingers; young participants performed 25 trials and old participants performed 20 trials
Olafsdo et al. (2007a; b)	8	Male and female (*n* = 10) healthy adults (27 ± 4 years); right-handed	Male and female (*n* = 10) healthy adults (77 ± 4 years); right-handed	Multi-finger force kinetic	UCM model included force variability among four fingers (elemental variable) to stablise a performance variable (total force); Δ*V* was reported	The subjects were asked to produce a ramp pattern of force from 0–10% of MVC over 5 s by pressing down with four fingers under reaction time and self-paced conditions; young participants performed 15 trials and old participants performed 20 trials; *only data from self-paced condition was used in this study*
Park et al. (2011)	8	Male and female (*n* = 7) healthy adults (29.8 ± 2.27 years); right- handed	Male and female (*n* = 7) healthy adults (79.4 ± 4.31 years); right-handed	Multi-finger force kinetic	UCM model included force variability among four fingers (elemental variable) to stablise a performance variable (total force); *V*_UCM_, *V*_ORT_ and Δ*V* were reported	Participants were instructed to press down four fingers and produce a specific total force (MVC%); 20 trials were completed
Park et al. (2016)	6	Male and female (*n* = 14) healthy adults (21.1 ± 1.3 years); right- handed	Male and female (*n* = 14) healthy adults (78.5 ± 4.63 years); right-handed	Multi-finger force kinetic	UCM model included force variability among two fingers (elemental variable) to stablise a performance variable (total force); *V*_UCM_, *V*_ORT_ and Δ*V* were reported	Participants were asked to produce a specific target force with 2 fingers under mechanical constraint and normal conditions; 10 trials per condition were completed; *Only normal condition was used in this study*
Shim et al. (2004)	8	Male and female (*n* = 12) healthy adults (26.2 ± 2.8 years); right- handed	Male and female (*n* = 12) healthy adults (82.6 ± 5.3 years); right-handed	Multi-finger force kinetic	UCM model included force variability among four fingers (elemental variable) to stablise a performance variable (total force); *V*_UCM_, *V*_ORT_ and Δ*V* were reported	Participants were instructed to track a line by producing a specific force (20MVC%) by pressing four fingers within 6 s; 20 trials were completed
Shinohara et al (2004)	8	Male and female (*n* = 12) healthy adults (28.9 ± 4.4 years); right-handed	Male and female (*n* = 12) healthy adults (82.1 ± 8 years); right-handed	Multi-finger force kinetic	UCM model included force variability among four fingers (elemental variable) to stablise a performance variable (total force); Δ*V* was reported	Participants were instructed to reach a target force (30 MVC%) with pressing four fingers; 12 trials were completed
Singh et al (2013)	6	Male and female (*n* = 8) healthy adults (25.8 ± 3.3 years); right-handed	Male and female (*n* = 8) healthy adults (74.4 ± 4.5 years); right-handed	Multi-finger force kinetic	UCM model included force variability among four fingers (elemental variable) to stablise a performance variable (total force); *V*_UCM_, *V*_ORT_ and Δ*V* were reported	Participants were instructed to reach a target force (40MVC%) by pressing four fingers; 2 trials were completed
Skm et al (2012)	7	Male and female (*n* = 9) healthy adults (27.3 ± 1.2 years); right- handed	Male and female (*n* = 9) healthy adults (77.6 ± 0.6 years); right-handed	Multi-finger force kinetic	UCM model included force variability among four fingers (elemental variable) to stablise a performance variable (total force); Δ*V* was reported	Participants were required to rotate a handle from neutral to a target position (30 degrees) in slow and fast speed; 24 trials were completed; *Only fast speed condition was selected in this study*
Solnik et al. (2012)	8	Male and female (*n* = 8) healthy adults (25.7 ± 5.1 years); right- handed	Male and female (*n* = 8) healthy adults (77.1 ± 5.2 years); right-handed	Multi-finger force kinetic	UCM model included internal force variability among four fingers (elemental variable) to stablise a performance variable (total force); Normal force, Tangential foce and ratio were	Participants were given a handle to hold with finger tips in air and vertical to the floor for 3 s; different types of grip were used; 36 trials were completed
Verrel et al. (2012)	9	Male and female (*n* = 12) healthy adults (25.5 ± 2.2 years); right- handed	Male and female (*n* = 12) healthy adults (73.4 ± 2 years); right-handed	Multi-joint kinematic	UCM model included a 11 Dofs elemental variable (clavicle, shoulder, elbow, wrist and fingers) and one performance variable (finger tip position); GEV , NGEV and UCM index were reported	Participants had to point targets with the right index finger; no reaction time requirement; targets were mounted on a stand (Up/Low); 3 practice conditions (blocked/alternating/random); 40 trials per condition were completed
Wu et al. (2013)	7	Male and female (*n* = 16) healthy adults (26.9 ± 4.9 years); right- handed	Male and female (*n* = 10) healthy adults (76.1 ± 5.6 years); right-handed	Multi-finger force kinetic	UCM model included internal force variability among four fingers (elemental variable) to stablise a performance variable (total force); *V*_UCM_ , *V*_ORT_ and Δ*V* were reported	Participants were instructed to reach a target force (40 MVC%) by pressing four fingers to match a target force template; 12 trials were completed
Xu et al. (2013)	8	Female healthy (*n* = 9) adults (25.6 ± 3.9 years); right-handed	Female healthy (*n* = 9) adults (61.8 ± 4.5 years); right-handed	Multi-joint kinematic	UCM model included a 7 Dofs elemental variable (shoulder, elbow and wrist) and one performance variable (hand position); *V*_UCM_, *V*_ORT_ and Δ*V *were reported	A simulated assembly task was used that required reach, grasp and release movements; 4 sessions (20 min each) were completed

### Quality assessment

The mean of quality score in all studies was 7.4 ± 0.54 (Table 1), in kinematic multi-joint studies was 7.2 (± 1.5) and in kinetic multi-finger studies was 7.61 (± 0.96). There were two studies with a low score (6: Park et al. 2007; Singh et al. 2013) and three studies with a highest score (9: Gorniak et al. 2011; Kapur et al. 2010a, b; Verrel et al. 2012). The main methodological issues in the selected studies were inadequate sample definition (item 1).

### Meta-analysis

#### Synergy index

**Fig. 2:**
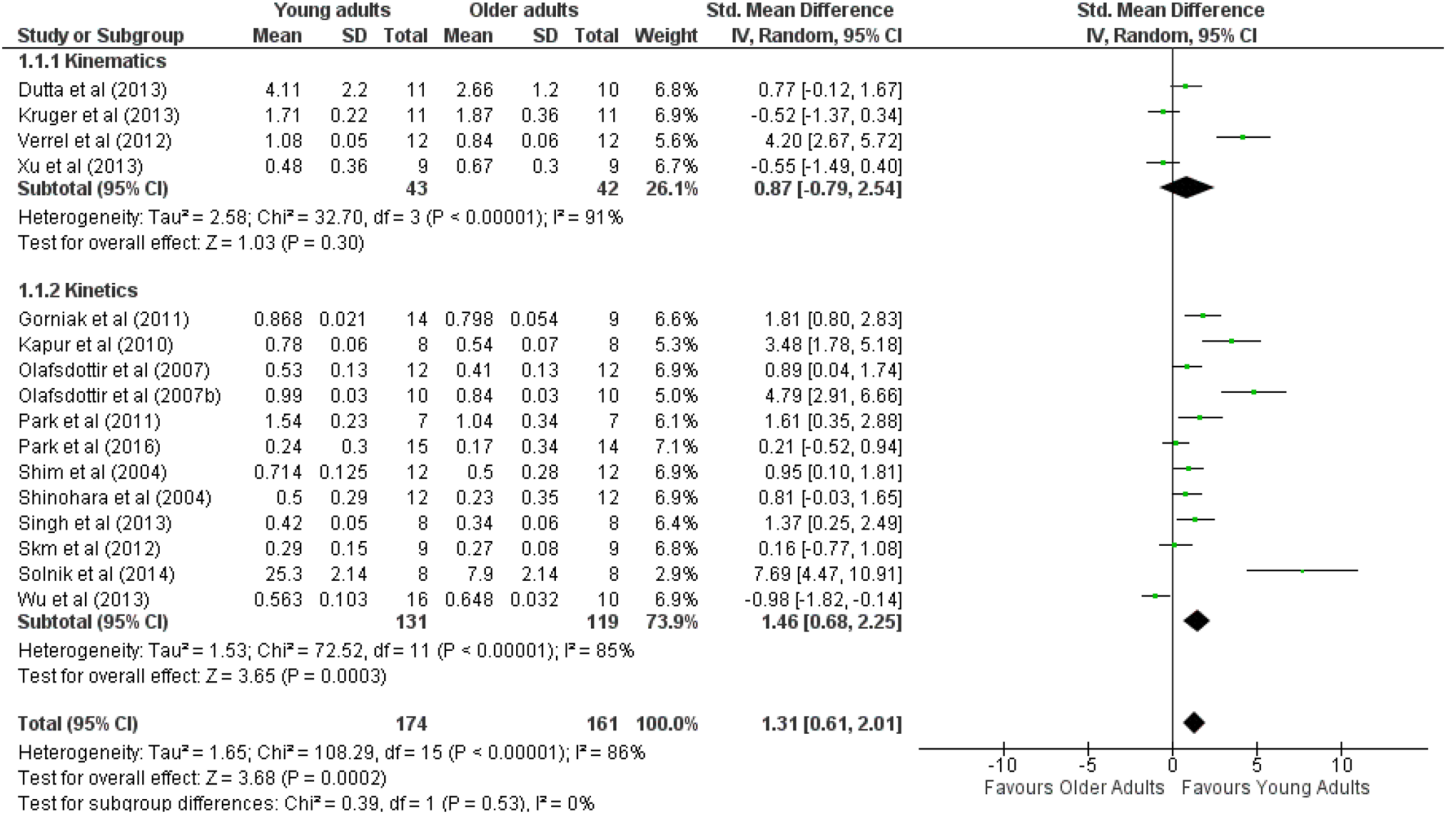
Forest plot comparing the motor synergies index between young and older adults in kinetics synergies and kinematics synergies tasks

In total, 174 young adults and 161 older adults participated in the selected studies (See Fig. 2). The results of meta-analysis have shown that synergy index was higher in younger adults, regardless of the type of synergy (ES_mean_ = 1.31, *Z* = 3.68, *p* < 0.05). Cochran *Q*^2^ results showed high heterogeneity (*Q*^2^ = 1.65, *I*^2^ = 86%) among studies.

There was a non-significant main effect of age group (ES_mean_ = 0.87, *Z* = 1.03, *p* > 0.05) on synergy index in multi-joint tasks. The results of Cochran *Q*^2^ have shown high heterogeneity (*Q*^2^ = 2.58, *I*^2^ = 91%) among studies. Only one study with a significant effect size showed stronger kinematic synergy in young adults relative to older adults (Verrel et al. 2012).

Furthermore, the young adults demonstrated significantly stronger indices of synergy (ES_mean_ = 1.46, *Z* = 3.65, *p* < 0.05) in multi-finger tasks than older adults. The results of Cochran *Q*^2^ have shown high heterogeneity (*Q*^2^ = 1.53, *I*^2^ = 85%) among studies. Most studies on multi-finger task showed a significant and large effect size in young adults (ESs range between 0.89 and 7.69). Only one study (Wu et al. 2013) showed a significant and large effect size in older adults (ES = − 0.98).

**Fig. 3:**
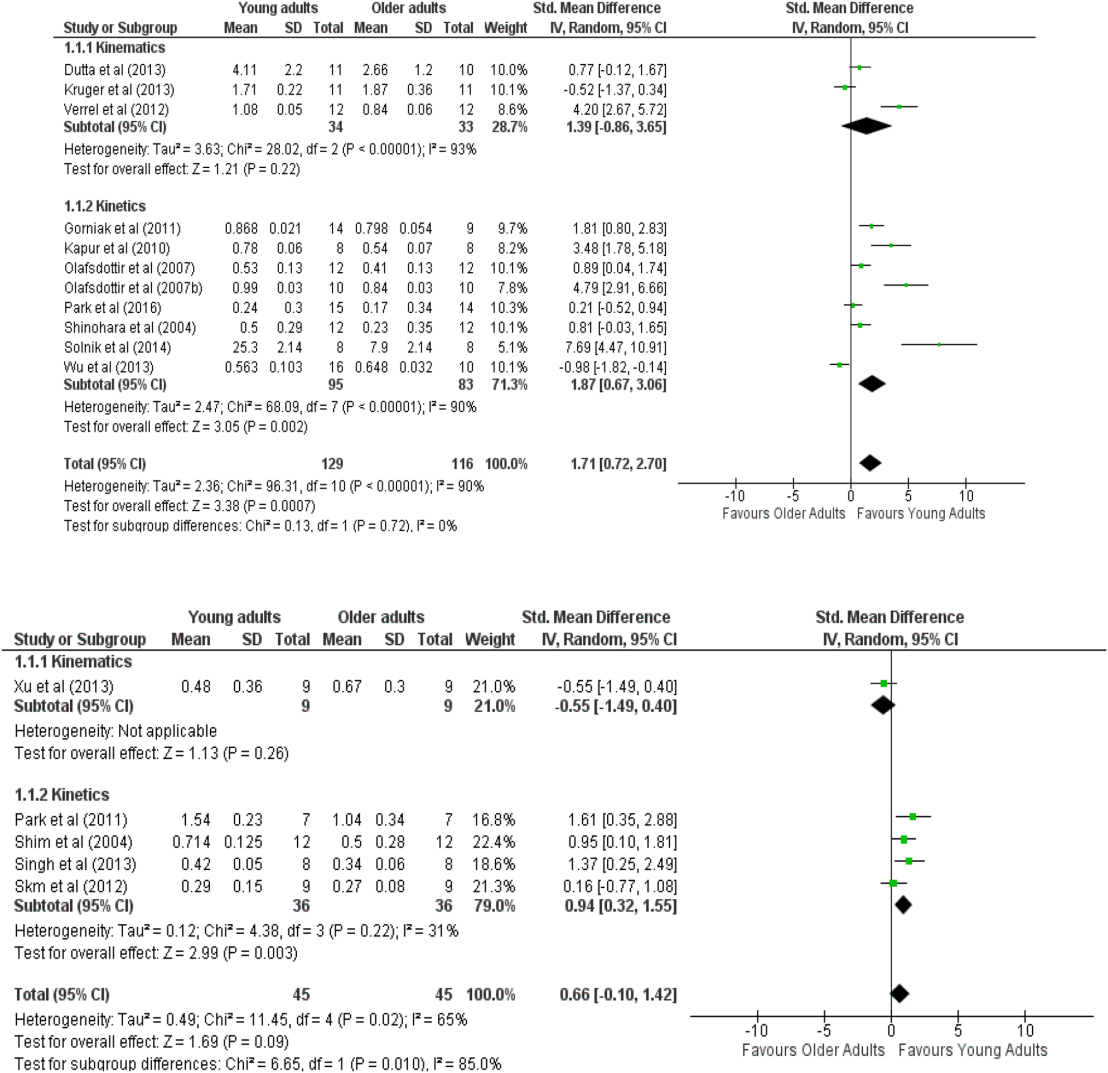
Forest plot comparing the motor synergies index between young and older adults in kinetics synergies and kinematics synergies tasks based on the measurement units. Δ*V* for kinetics and ratio for kinematics (top) and Δ*V*z for both kinetics and kinematics (bottom)

Figure 3 shows the results based on different units of measure. The results failed to show a significant main effect of age group on kinematic synergies in multi-joint tasks with ratio (ES_mean_ = 1.39, *Z* = 1.21, *p* > 0.05) and Δ*V*z (ES_mean_ = − 0.55, *Z* = 1.13, *p* > 0.05) unit of measurement. The main effect of age group was significant on kinetic synergies in multi-finger tasks when the unit of measurement was reported as Δ*V* (ES_mean_ = 1.87, *Z* = 3.38, *p* < 0.05) and Δ*V*z (ES_mean_ = 0.94, *Z* = 2.99, *p* < 0.05).

#### Goal equivalent variability

**Fig. 4:**
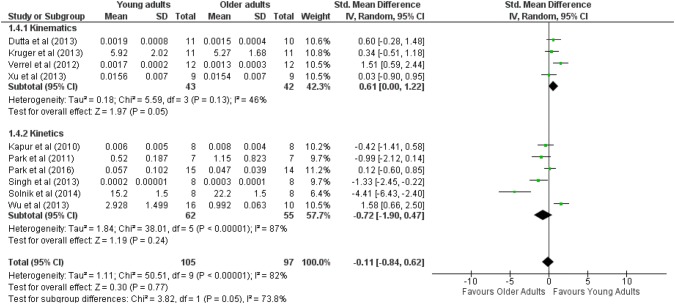
Forest plot comparing the GEV between young and older adults in kinetics synergies and kinematics synergies tasks

The pool effect size was not significant for GEV, regardless of the type of synergies (ES_mean_ = − 0.11, *Z* = 0.3, *p* > 0.05). Cochran *Q*^2^ results have shown high heterogeneity (*Q*^2^ = 1.11, *I*^2^ = 82%) among studies (see Fig. 4).

A moderate effect has shown on synergies in multi-joint tasks and younger adults exhibited more GEV (ES_mean_ = 0.61, *Z* = 1.97, *p* < 0.05). The results of Cochran *Q*^2^ have shown low heterogeneity (*Q*^2^ = 0.18, *I*^2^ = 46%) among studies. Only one study showed a significant effect size (Verrel et al., 2012; ES = 1.51); however, all studies reported a low to moderate effect and younger adults exhibited more GEV than older adults.

There was no significant main effect of ageing (ES_mean_ = − 0.72, *Z* = 1.19, *p* > 0.05) on kinetic synergies in multi-finger tasks. The results of Cochran *Q*^2^ have shown high heterogeneity (*Q*^2^ = 1.84, *I*^2^ = 87%) among studies. Only one study showed that in young adults the GEV was higher than older adults (Wu et al. 2013), whereas two studies showed that older adults had higher GEV on kinetic synergy (Singh et al. 2013; Solnik et al. 2012).

#### Nongoal equivalent variability

**Fig. 5:**
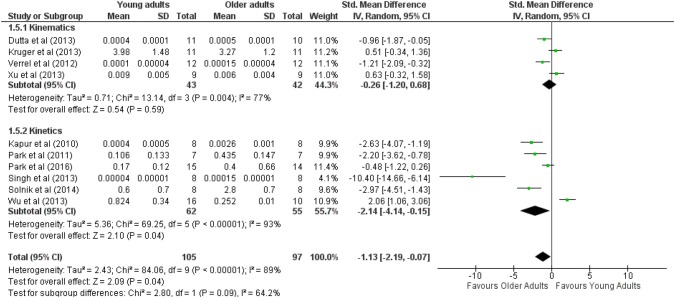
Forest plot comparing the NGEV between young and older adults in kinetics synergies and kinematics synergies tasks

The pool effect size was significant and higher in older adults (ES_mean_ = − 1.13, *Z* = 2.09, *p* < 0.05). Cochran *Q*^2^ results have shown high heterogeneity (*Q*^2^ = 2.43, *I*^2^ = 89%) among studies (see Fig. 5).

The results on kinematic synergies in multi-joint tasks failed to show a significant main effect of age group (ES_mean_ = − 0.26, *Z* = 0.54, *p* > 0.05). The results of Cochran *Q*^2^ have shown high heterogeneity (*Q*^2^ = 0.71, *I*^2^ = 77%) among studies. Only two studies showed a significant effect size that was higher in older adults (Verrel et al. 2012; Dutta et al. 2013).

The older adults demonstrated greater NGEV than young adults (ES_mean_ = − 2.14, *Z* = 2.1, *p* < 0.05). The results of Cochran *Q*^2^ have shown high heterogeneity (*Q*^2^ = 5.36, *I*^2^ = 93%) among studies. The majority of studies on synergies in multi-finger tasks showed a significant and large effect size and older adults exhibited more NGEV (ESs range between − 2.2 and − 10.4). Only one study (Wu et al. 2013) showed a significant large effect size and greater NGEV in young adults (ES = 2.06).

## Discussion

Motor synergies are affected by ageing due to the sensory-motor changes in the neuromusculoskeletal system (Cole et al. 1999). The main aim of this study was to compare the motor synergy index and its variance components (GEV and NGEV) between young and older adults. The results of this meta-analysis showed that the young adults have significantly greater synergies in multi-finger tasks. The stronger synergies were mainly due to the lower NGEV in younger adults. Despite the higher but non-significant GEV in the young adults, it was not the main determinant of kinematic synergies in multi-joint tasks. The significant main effect of ageing on multi-finger synergies was independent from the unit of measurements (Δ*V*, Δ*V*_Z_) that was used differently in previous studies. The following sections discuss the age-related changes in two main areas: multi-joint and multi-finger tasks.

### Older adults preserve kinematic synergies in multi-joint tasks

The results of this study showed that there was no significant difference between young and older adults in manipulative tasks that require kinematic synergies in multi-joint tasks. There are several potential explanations for the preservation of kinematic synergies in older adults.

The motor system might perform visually guided tasks such as reaching without compromising motor flexibility (Cressman and Henriques 2010) in both young and older adults. The alternative explanation is visuomotor adaptation that requires transformation, modification and integration of information from the object with respect to the hand position at initial and during the reaching (Buch et al. 2003). The results of some studies showed no age-related deficits on visuomotor adaptation in manual reaching tasks (Roller et al. 2002; Buch et al. 2003). Sensorimotor adaptation can be improved by two types of process: recalibration and strategic control (Redding 1996). Recalibration implies that the sensory input and motor outputs are re-aligned or the internal model is modified. Strategic control implies that the performer uses visual feedback to correct the movement.

Ageing appears to affect the strategic control process and the recalibration is not impaired (Bock 2005). The possible explanations regarding the adverse effects of ageing on strategic control process were cognitive dysfunction in older adults due to shrinkage of the prefrontal cortex (Raz et al. 1997) and associated reduction of dopamine activity (Volkow et al. 1998). The plausible reason for an intact recalibration process in older adults could be a repetition of a same response that is learned during adaptation and preservation, which is predominant with advancing age (Nagahama et al. 1997). In the current study, we found that the ES was significant only in one study and was higher in young adults (Verrel et al. 2012). It seems that older adults in this study adapted gradually to the constraints of reaching tasks using proprioceptive recalibration (Cressman et al. 2010) rather than motor flexibility.

Although non-significant, effect sizes in three studies indicated a trend towards greater GEV in young adults (Dutta et al. 2013; Krüger et al., 2013; Xu et al. 2013), which could be related to the nature of the task variable (single variable task) used in each of the three studies. Multiple variable tasks (direction and pace) were beneficial for older adults (Lee et al. 2007), whereas in Verrel et al. (2012), the single-variable task (fix target) was a disadvantage for this age group.

Other task-specific control determinants that might associate with the lack of age differences on reaching kinematics are biomechanical constraints such as movement velocity and hand path. A recent study showed that movement time and velocity during reaching are not different between the young and older adults and cannot be attributed to any changes in synergy components (Xu et al. 2013). Furthermore, age-related changes in hand function are evident in the stabilisation of hand orientations rather than hand position (Krüger et al. 2013). This suggests that older adults could adapt joint configurations differently in tasks with multiple as opposed to single constraints.

Preservation of kinematic synergies in multi-joint tasks in older adults might be related to the unique features of synergies in this group. For example, Reisman and Scholz (2003) showed that, in people with stroke, the strength of motor synergies to stabilise the path of the paretic hand during reaching is similar to able-bodied individuals. It seems that the emerged synergies among elemental variables instead of a reduction in the trial-to-trial variability (error compensation) play a significant role in controlling the average contribution of elemental variables (sharing synergies). The shared feature of motor synergies (Latash, et al. 2007) might explain how older adults were able to coordinate the elemental variables same as young adults.

The current meta-analysis did not reveal a significant difference on overall ES between the age groups for NGEV. The studies with a significant ES (Dutta et al. 2013; Verrel et al. 2012) showed that NGEV was greater in older adults (See Fig. 4). The greater NGEV was associated with a slower movement speed (Scholz et al. 2011).

However, these results and conclusions should be interpreted with caution, because only four studies have been used in the current study.

### Ageing reduces the strength of kinetic synergies in multi-finger tasks

The effects of ageing on synergies in multi-finger tasks were remarkable, indicated by both overall mean ES (1.14) and individual studies ESs (range between 0.89 and 7.69). Several studies (Kapur et al. 2010a, b; Park et al. 2011; Singh et al. 2013; Solnik et al. 2012) demonstrated that an increase in NGEV accounts for the changes in finger synergies. Only Wu et al. (2013) showed a greater effect size and lower NGEV in older adults.

Ageing is accompanied by neural and structural changes in the CNS (Brooks and Faulkner 1994; Schieber 2001), and weakened synergies among fingers could be associated with these changes (Latash et al. 2002a, b). Sensory and motor neuron changes at different levels of CNS have been shown to be mechanisms responsible for losing motor synergies in older adults (Latash and Anson 2006). Studies in people with Parkinson's disease further suggest that changes in finger coordination may be a common feature of subcortical disorders (Jo et al. 2015). Furthermore, as covariance of shared force among fingers is reduced in older adults, older adults shift from more complex and synergic control to the more element-based and less redundant control due to the progressive death of neurons at different levels (Gorniak et al. 2011). It also demonstrated that the ageing had adverse effects on the number of motor units in hand muscles that lead to emergence of larger and slower motor units (Grabiner and Enoka 1995), reinnervation of muscle bundles, atrophy in muscle fibres and decrease in a total number of fibres (Thompson 2009) that result in a reduction in muscle force and deterioration of hand function (Cole et al. 1999).

Losing finger synergies has negative consequences on the older adults' experience of tasks in daily life. For example, motor synergies are organised to stabilise the net moment of force produced by the fingers which help to stabilise the rotational action of the hand (Zatsiorsky et al. 2000). Because rotational actions are used frequently during ADLs that involve pressing and prehensile tasks (Shim et al. 2004), age-related changes in finger synergies could affect the older adults’ ability to perform, and experience of, tasks in daily life. Another line of research on the association between the nature of tasks and reductions in the strength of finger synergies is related to the application of force during grasping. Modelling work indicates that rapidly changing multi-finger force production increases the NGEV that corresponds to the destabilisation of the total force (Goodman et al. 2005). Additionally, it seems that the age differences in finger synergies become, to some extent, smaller when the nature of the task is more repetitive and less complex—such as simple tasks versus dual tasks (Park et al. 2016).

Generally, the weaker finger synergies following ageing—and in patients with Parkinson's disease and Multiple Sclerosis—could reflect lower stability of performance variable—grasping force—and delayed adjustments in preparation for quick action (Jo et al. 2015, 2017). More specifically, it seems that older people have two limitations in employing the motor synergies in multi-finger tasks: lower accuracy and lower stability. Reduced grip force accuracy could be related to deterioration of cutaneous sensory functioning (Johansson 1996) that alters the amount of grip force that is required to control the slipping -safety margins (Kinoshita and Francis 1996). This dysfunction is more apparent in the grip tasks that varied in terms of friction, external loadings and reliability of anticipatory control mechanisms (Cole et al. 1999). The low steadiness could be explained by the subclinical dysfunctions in the nervous system (Beijersbergen et al. 2013; Faulkner et al. 2007; Thompson 2009) that contribute to the sensory-motor synchronisation and the muscle force (Larsson and Ansved 1995). The elderly people are impaired in their ability to coordinate individual digit forces and moments to ensure stable performance with respect to the force/moment production tasks (Shim et al. 2004). Inability to maintain the performance variable (grip force) has been identified as an underlying mechanism to explain the fine-motor control deterioration in older adults (Grabiner and Enoka 1995; Lindberg et al. 2009).

## Conclusion

The results of this study showed that the structural and functional changes following ageing in the CNS and muscular system have significant negative impacts on kinetic synergies in multi-finger tasks but not kinematic synergies in multi-joint tasks. The age-related changes in kinetic synergies could negatively affect the strategy for the recruitment of fingers to stabilise the total finger force in safe and firm grasp tasks. Furthermore, the weaker kinetic synergies are related to increased NGEV in older adults. It seems that the adopting an element-based control strategy reduces the cooperation among the fingers to achieve the task goal, amplifying performance variability.
